# Specific Patterns of White Matter Alterations Help Distinguishing Alzheimer's and Vascular Dementia

**DOI:** 10.3389/fnins.2018.00274

**Published:** 2018-04-25

**Authors:** Fulvia Palesi, Andrea De Rinaldis, Paolo Vitali, Gloria Castellazzi, Letizia Casiraghi, Giancarlo Germani, Sara Bernini, Nicoletta Anzalone, Matteo Cotta Ramusino, Federica M. Denaro, Elena Sinforiani, Alfredo Costa, Giovanni Magenes, Egidio D'Angelo, Claudia A. M. Gandini Wheeler-Kingshott, Giuseppe Micieli

**Affiliations:** ^1^Department of Physics, University of Pavia, Pavia, Italy; ^2^Brain Connectivity Center, IRCCS Mondino Foundation, Pavia, Italy; ^3^Department of Electrical, Computer and Biomedical Engineering, University of Pavia, Pavia, Italy; ^4^Brain MRI 3T Mondino Research Center, IRCCS Mondino Foundation, Pavia, Italy; ^5^Neuroradiology Unit, IRCCS Mondino Foundation, Pavia, Italy; ^6^Department of Brain and Behavioral Sciences, University of Pavia, Pavia, Italy; ^7^Alzheimer's Disease Assessment Unit, Laboratory of Neuropsychology, IRCCS Mondino Foundation, Pavia, Italy; ^8^Scientific Institute H. S. Raffaele, Milan, Italy; ^9^Department of Emergency Neurology, IRCCS Mondino Foundation, Pavia, Italy; ^10^Queen Square MS Centre, UCL Institute of Neurology, Faculty of Brain Sciences, University College London, London, United Kingdom

**Keywords:** Alzheimer's disease, vascular dementia, DTI, splenium of corpus callosum, genu of corpus callosum, parahippocampal gyri, thalamic radiations

## Abstract

Alzheimer disease (AD) and vascular dementia (VaD) together represent the majority of dementia cases. Since their neuropsychological profiles often overlap and white matter lesions are observed in elderly subjects including AD, differentiating between VaD and AD can be difficult. Characterization of these different forms of dementia would benefit by identification of quantitative imaging biomarkers specifically sensitive to AD or VaD. Parameters of microstructural abnormalities derived from diffusion tensor imaging (DTI) have been reported to be helpful in differentiating between dementias, but only few studies have used them to compare AD and VaD with a voxelwise approach. Therefore, in this study a whole brain statistical analysis was performed on DTI data of 93 subjects (31 AD, 27 VaD, and 35 healthy controls—HC) to identify specific white matter patterns of alteration in patients affected by VaD and AD with respect to HC. Parahippocampal tracts were found to be mainly affected in AD, while VaD showed more spread white matter damages associated with thalamic radiations involvement. The genu of the corpus callosum was predominantly affected in VaD, while the splenium was predominantly affected in AD revealing the existence of specific patterns of alteration useful in distinguishing between VaD and AD. Therefore, DTI parameters of these regions could be informative to understand the pathogenesis and support the etiological diagnosis of dementia. Further studies on larger cohorts of subjects, characterized for brain amyloidosis, will allow to confirm and to integrate the present findings and, furthermore, to elucidate the mechanisms of mixed dementia. These steps will be essential to translate these advances to clinical practice.

## Introduction

Dementia is one of the major causes for personal and financial issues affecting elderly people; indeed, dementia incidence rate is increasing because aging population is constantly rising. Alzheimer disease (AD) and vascular dementia (VaD) together represent the majority of dementia cases (Rizzi et al., 2014).

AD is a primary degenerative disorder associated to ß-amyloid and tau deposition, characterized by progressive deterioration in memory, language, perceptual skills, attention, orientation, and problem solving. In older subjects and in the presence of cerebral amyloid angiopathy, AD patients can also present a certain degree of cerebrovascular lesions contributing to a mixed type of dementia (Attems and Jellinger, 2014). VaD is a heterogeneous disease where dementia is secondary to the development of a cerebrovascular lesion load, which is *per se* sufficient to determine dementia without primary degenerative changes. The entity of VaD impairment is reported to be proportional to number, location, and extent of cerebrovascular lesions (Suri et al., 2014). The cognitive impairment is dominated by executive/frontal and semantic memory dysfunctions but the neuropsychological profile can overlap with that of AD (Reed et al., 2007). Because in VaD vascular risk factors are currently treatable, a more certain differentiation from AD in the early stages of cognitive impairment would imply better patient management.

MRI studies have contributed to differential diagnosis identifying different alterations associated to the type of dementia. In AD cortical atrophy mainly involves temporal and parietal lobes, especially temporomesial regions and the precuneus/posterior cingulate cortex; furthermore microstructural abnormalities extend beyond the primarily affected cortical regions, involving degeneration of white matter (WM) bundles even in early stages of the disease (Villain et al., 2010). An indirect relation between WM involvement and tau accumulation in CSF, which is a marker of neurodegeneration and disease progression, has also been shown (Agosta et al., 2014). In VaD, instead, the regional pattern of cerebrovascular lesions varies among different VaD subtypes (Jellinger, 2013; O'Brien and Thomas, 2015). In the most common form, small vessel disease leads to subcortical vascular dementia with multifocal lesions observed on qualitative MRI predominantly in deep gray matter (lacunae and gliosis in basal ganglia and thalami), and mainly in frontal periventricular and subcortical WM (leukoariosis and more rare lacunae) (Savoiardo and Grisoli, 2001; Vitali et al., 2008) (Table 1). Previous studies have tried to distinguish between neurodegenerative and vascular dementia using diffusion-based techniques (Altamura et al., 2016; Goujon et al., 2018) but discordant results have been obtained. Both Goujon et al. (2018) and Altamura et al. (2016) used a ROI approach on apparent diffusion coefficient (ADC) maps; the former study demonstrated lower ADC in frontal and parietal deep WM regions in AD with respect to subcortical changes in VaD; the latter study, instead, reported correlations between WM volume and ADC of the corpus callosum, but no differences in regional ADCs in WM between AD and VaD patients were found. A further study (Baykara et al., 2016) has developed a novel imaging marker for small vessel disease that was linked to vascular but not neurodegenerative disease. Therefore, it is essential to continue developing quantitative *in vivo* biomarkers, specific for AD or VaD to support differential diagnosis, to investigate disease progression at single patient level, and to monitor the effect of therapeutic interventions in pharmacological trials.

**Table 1:** Summary of the main features of Alzheimer's disease and vascular dementia.

	**Alzheimer**	**Vascular dementia**
Risk factors	APOE, diabetes	Hypertension, diabetes, dyslipidemia, atheromatosis
Etiology	Amyloid plaques and neurofibrillary tangles	Vascular lesions
Clinical evolution	Progressive	Stepwise
Neuropsychological profile	Memory impairment (encoding)	Executive/frontal and semantic dysfunction
Neuroimaging	Medial temporal lobe atrophy, amyloid PET positivity, FDG hypometabolism in parieto-temporal regions	White matter lesions, lacunae, leukoaraiosis

Advances in neuroimaging over the past decades have indicated that investigating structural and functional brain alterations in humans *in vivo* is now possible. The role of advanced MRI techniques in diagnosis has become more prominent and inclusion of quantitative metrics has been recommended as part of the diagnostic pipeline (McKhann et al., 2011). In particular, diffusion tensor imaging (DTI) is a MRI technique sensitive to water molecules diffusivity in tissues, and DTI-derived parameters quantitatively describe such movement and highlight microstructural WM changes in term of diffusion properties of tissue (Acosta-Cabronero et al., 2010; Meoded et al., 2017). Fractional anisotropy (FA) and mean diffusivity (MD) are the most reported DTI-derived parameters to characterize dementia. The former reflects anisotropy properties of WM tissues, while the latter reflects the magnitude of water diffusion (Smith et al., 2007; Chua et al., 2008). Several studies have reported reduced FA and increased diffusivity in specific WM regions mainly involving the limbic system and posterior-anterior connections in AD (Acosta-Cabronero et al., 2010; Tu et al., 2017; Goujon et al., 2018), while VaD is characterized by spread microstructural alterations, i.e., increased MD and decreased FA, across all WM regions except occipital areas (Kim et al., 2015; Ostojic et al., 2015).

Further information can be gathered from the preferential direction of water displacement and the orthogonal components, as expressed by axial diffusivity (AxD) and radial diffusivity (RD) (Wheeler-Kingshott and Cercignani, 2009; Acosta-Cabronero et al., 2010; Shu et al., 2011; Bosch et al., 2012; van Bruggen et al., 2012). Moreover, the pattern of microstructural WM involvement, more posterior in AD and more anterior in VaD, has been investigated with DTI metrics using a-priori defined regions of interest (Sugihara et al., 2004; Ostojic et al., 2015).

Several methods have been introduced aiming at improving the objectivity and interpretability of DTI metrics, and Tract-Based Spatial Statistics (TBSS) has been developed specifically for whole brain voxel-wise analysis of white matter changes. TBSS is an automated voxel-wise analysis method that gives the opportunity to compare the whole WM in groups of subjects, without any predefined regions of interest (Smith et al., 2006). One of the main issues of voxel-wise analysis on subjects with dementia lies in the presence of brain atrophy, which leads to misalignment to a reference template. Using TBSS this problem is minimized since from normalized FA images of each subject only the “main WM skeleton” is extracted and analyzed (i.e., the center of all tracts common to all subjects).

TBSS has been largely used to assess AD alterations (Damoiseaux et al., 2009; Acosta-Cabronero et al., 2010; Shu et al., 2011; Bosch et al., 2012) but only two studies (Zarei et al., 2009; Kim et al., 2015) have used this method for investigating differences in terms of FA and MD between AD, VaD and elderly healthy controls (HC). Zarei et al. (2009) found that the most important combination of parameters to differentiate AD and VaD was one obtained by integrating FA value of the forceps minor with the Fazekas score. This finding confirmed the importance of segmenting the corpus callosum and searching for changes in transcallosal prefrontal tracts of patients with different types of dementia. Furthermore, Kim et al. (2015) showed that microstructural alterations affected all WM regions of the brain apart from few specific areas in VaD, while decreased FA was mainly found in the fronto-parietal WM regions in AD patients. These results support the hypothesis that VaD and AD patients have different topography, nevertheless specific patterns of microstructural alterations were not identified as able to distinguish between VaD and AD.

The main goals of the present study were: (i) to identify specific patterns of WM alterations for AD and VaD by investigating changes of multiple DTI-derived parameters (FA, MD, AxD, RD) with the TBSS pipeline, and (ii) to assess the power of the identified patterns of alterations in discriminating between AD and VaD patients.

## Materials and methods

### Subjects

A total of 93 subjects including 31 AD (age (73 ± 7) years, 12 females (F), Mini Mental State Examination (MMSE) score = 16 ± 6), 27 VaD (age (77 ± 8) years, 21 F, MMSE = 18 ± 4) and 35 HC (age (69 ± 10) years, 17 F, MMSE = 28 ± 1) were enrolled in this study. This study was carried out in accordance with the Declaration of Helsinki with written informed consent from all subjects. The protocol was approved by the local ethic committee of the IRCCS Mondino Foundation. Inclusion criteria for patients were: clinical diagnosis of dementia on the basis of DSM 5 criteria (American Psychiatric Association, 2013), Mini-Mental State Examination (MMSE) score (Folstein et al., 1975) below 24 and age above 60 years. All patients who presented with at least one of the following criteria were excluded: epilepsy or isolated seizures; major psychiatric disorders over the previous 12 months; pharmacologically treated delirium or hallucinations; ongoing alcoholic abuse. Exclusion criteria were also acute ischemic or hemorrhagic stroke, known intracranial lesions, and systemic causes of subacute cognitive impairment (Geschwind et al., 2008). Patients with dementia occurring after acute ischemic or hemorrhagic stroke were excluded, while those patients presenting WM alterations due to small vessel disease were included. Healthy controls were enrolled on a voluntary basis among subjects attending to a local third age university (University of Pavia, Information Technology course) or included in a program on healthy aging (Fondazione Golgi, Abbiategrasso, Italy). Diagnosis of AD was made according to the criteria of the National Institute of Neurological and Communicative Disorders and Stroke and Alzheimer's Disease and Related Disorders Association (NINCDS-ADRDA) workgroup (McKhann et al., 2011). For definition of vascular dementia (VaD) the diagnostic criteria of the National Institute of Neurological Disorders and Stroke – Association Internationale pour la Recherche et l'Enseignement en Neurosciences (AIREN) criteria (Roman et al., 1993) were used.

### Clinical and neuropsychological examination

All 93 subjects underwent clinical and neuropsychological standardized testing for investigating the global cognitive status (MMSE) and the following main cognitive domains: attention (attentive matrices, trail making test A and B, Stroop test), memory (digit span, verbal span, Corsi block-tapping test, logical memory, Rey–Osterrieth complex figure recall, Rey 15 item test), language (phonological and semantic verbal fluency), executive function (Raven's matrices, Wisconsin card sorting test, frontal assessment battery), and visuo-spatial skills (Rey–Osterrieth complex figure) (Carlesimo et al., 1996; Giovagnoli et al., 1996; Laiacona et al., 2000; Caffarra et al., 2002; Appollonio et al., 2005; Amato et al., 2006; Bianchi and Dai Prà, 2008). Age- and education-corrected scores were calculated from the raw scores and transformed into equivalent scores, ranging from 0 (pathological) to 4 (normal). This standard procedure ensures that all final equivalent scores are comparable between groups even in the presence of differences in age and years of education. For each cognitive domain, a weighted score was calculated as the average value of the equivalent scores of all tests belonging to that specific cognitive domain (de Groot et al., 2000; van Dijk et al., 2008). These scores were then used in the statistical analysis. Clinical classification of AD or VaD was performed according to the above mentioned criteria and was further refined by excluding patients with mixed dementia according to the Hachinski scale (HS) (Hachinski et al., 1975) with pathology-validated cut-offs (Moroney et al., 1997): pure vascular (HS ≥ 7), pure degenerative (HS ≤ 4), and mixed dementia (HS 5 and 6). Semi-quantitative rating of vascular abnormalities was performed on radiological bases by evaluating WM leukoaraiosis according to the Fazekas scale (Fazekas et al., 1987) and deep gray/white matter lesions according to the “basal ganglia” (striatum, globus pallidus, thalamus, internal/external capsule, and insula) subscale of the age-related white matter changes (ARWMC) scale (http://stroke.ahajournals.org/content/32/6/1318, Wahlund et al., 2001).

### MRI acquisition

MRI data were acquired with a 3T Siemens Skyra scanner (Siemens, Erlangen, Germany). A DTI twice refocused SE-EPI sequence was acquired using the following parameters: TR = 10 s, TE = 97 ms, 70 axial slices with no gap, acquisition matrix = 122 × 122, 2 mm isotropic voxel; 64 volumes with non-collinear diffusion directions with b = 1,200 s/mm^2^ and 10 volumes with no diffusion weighting (b_0_ = 0 s/mm^2^). For anatomical reference a whole brain high-resolution 3D sagittal T1-weighted (3DT1w) scan was acquired using the following parameters: TR = 2,300 ms, TE = 2.95 ms, TI = 900 ms, flip angle = 9°, 176 sagittal slices, acquisition matrix = 256 × 256, in-plane resolution = 1.05 × 1.05 mm, slice thickness = 1.2 mm.

### DTI preprocessing

DTI data pre-processing was performed using FSL (FMRIB Software Library, http://fsl.fmrib.ox.ac.uk/fsl/fslwiki/). For each subject, the 10 b_0_ volumes were averaged and the obtained mean b_0_ image was merged with the 64 diffusion weighted volumes. DTI data were corrected for eddy current distortions and diffusion weighed volumes were aligned to the mean b_0_ image using eddy tool (FSL). A binary brain mask was obtained from the mean b_0_ volume using brain extraction tool (Smith et al., 2006), while DTIFIT was used to create FA, MD and AxD maps. RD maps were calculated implementing the RD formula with fslmaths (Acosta-Cabronero et al., 2010).

For each subject, the 3DT1w volume was segmented into WM and GM by using FAST (Zhang et al., 2001). The mean b_0_ image and along with the FA map was aligned to the respective 3DT1w volume using a full-affine registration (12 degrees-of-freedom, FLIRT) (Jenkinson et al., 2002). The mean FA value of the brain was then obtained from all voxels extracted from the FA map using a combined WM and GM mask.

### Tract-based spatial statistics (TBSS)

Tract-based spatial statistics was performed to investigate the voxel-wise distribution of FA, MD, AxD, and RD differences between groups. Such analysis was performed using the TBSS tool in the FMRIB software library (FSL, https://fsl.fmrib.ox.ac.uk/fsl/fslwiki). For a detailed description of the complete TBSS pipeline see Smith et al. (2006). Here a recap of the main steps is reported.

- The most representative subject, namely the FA map for which the smallest amount of average warping is necessary to align all other images to it, was identified as the *target image* on a sub-cohort of 15 randomly chosen subjects (5 for each group).
- FA maps of all subjects were nonlinearly aligned to the target image. Then all the aligned FA images were affine-transformed to the standard MNI152 template (1 × 1 × 1 mm^3^).
- The mean FA image across all subjects and its mean WM skeleton were created in the MNI152 space.
- The aligned subjects' FA images were projected onto the mean WM skeleton and the resulting maps were compared in a cross-subject statistical analysis (see next section).
- MD, AD and RD maps were also projected onto the mean WM skeleton using the same combination of transformations that was adopted for the FA maps alignment in the previous steps.

### Statistical analysis

Statistical tests were performed using SPSS software version 21 (IBM, Armonk, New York). Demographic, clinical and neuropsychological continuous data were tested for normality using a Shapiro-Wilk test. Age was compared between groups using a two-tailed Kruskall-Wallis test while gender was compared using a chi-squared test. Since the three groups resulted to be no age- and gender-matched, clinical and neuropsychological data were compared using a multivariate regression model with age and gender as covariates. To investigate whether age might produce an effect on our analysis, distributions of the age parameter in AD, VaD, and HC and their overlap were calculated and reported in Figure S1 in Supplementary Material. Two-sided *p* < 0.05 was considered statistically significant.

The voxel-wise statistics on the skeletonized images were performed using randomize (Winkler et al., 2014), an FSL tool for non-parametric permutation inference on neuroimaging data. The mean FA skeleton (thresholded at 0.2) was used as mask, the number of permutation was set at 5,000. Significance of difference between groups was corrected for multiple comparisons using the threshold-free cluster enhancement (TFCE) method and tested at *p* < 0.01. Age, gender and mean FA of the brain were used as covariates for the voxel-wise statistics to remove their influence on diffusion parameters and to avoid biased results. Comparisons were performed for all parameters (FA, MD, AxD, RD), testing differences between all groups of subjects.

Mean FA and MD values were extracted for 10 brain areas (or regions of interest - ROIs) that resulted as being particularly relevant from the TBSS statistical analysis. The areas were: bilateral parahippocampal tracts, cingulum bundles, thalamic radiations, and genu, anterior body, posterior body, and splenium of the corpus callosum. To identify the power of the selected 10 brain areas in discriminating between VaD and AD, a stepwise discriminant analysis was performed using the dementia subgroup (VaD or AD) as the dependent variable and the ROIs mean FA and MD values as the independent variables. Receiving Operating Characteristics (ROC) curves and corresponding areas under the curve were calculated to assess the sensitivity and specificity of the best discriminative variables. Furthermore, the relationship between the microstructural characteristics of the best discriminating brain regions and other radiological/clinical and neuropsychological scores was explored; this is a different question than locating regions where these features are related with microstructural alterations. Thus, a Pearson correlation analysis was performed between MRI derived parameters (i.e., total Fazekas score, ARWMC score), neuropsychological scores (Table 2) and the microstructural DTI-derived proprieties of the 10 selected ROIs in all patients, and in the AD and VaD groups separately. Two-sided *p* < 0.01 was considered statistically significant.

**Table 2:** Demographic and clinical evaluation.

	**HC (*****n*** = **35)**		**AD (*****n*** = **31)**		**VaD (*****n*** = **27)**		***p*-value**
	**Mean**	**(SD)**	**Mean**	**(SD)**	**Mean**	**(SD)**	
Age, years	69.43	(9.65)	72.52	(7.43)	76.67	(7.77)^*^^†^	0.002
Gender, % male	51.40		58.10		22.20	^*^^†^	0.015
Education, years	9.70	(3.99)	6.63	(3.30)^*^	5.52	(2.14)^*^	<0.001
MMSE	28.47	(1.51)	15.83	(6.37)^*^	17.90	(4.15)^*^	<0.001
Memory	2.99	(0.69)	0.88	(0.74)^*^	0.91	(0.53)^*^	<0.001
Attention	3.19	(0.80)	1.30	(0.85)^*^	0.88	(0.64)^*^	<0.001
Language	3.46	(0.82)	1.52	(1.30)^*^	1.28	(1.04)^*^	<0.001
Executive function	2.88	(0.81)	0.83	(1.01)^*^	0.53	(0.81)^*^	<0.001
Visuo-spatial skills	3.43	(1.17)	0.78	(1.48)^*^	0.62	(1.28)^*^	<0.001
Hachinski score	0.00	(0.00)	3.00	(0.86)^*^	8.27	(1.59)^*^^†^	<0.001
Fazekas score	0.00	(0.00)	2.48	(1.33)^*^	4.63	(1.52)^*^^†^	<0.001
AWMRC score	0.00	(0.00)	0.60	(0.71)^*^	1.27	(0.84)^*^^†^	<0.001

Demographic and clinical scores for healthy controls (HC), Alzheimer's disease (AD), and vascular dementia (VaD) groups. Values are expressed as mean (SD). MMSE, Mini Mental State Examination. p-values show statistically significant differences between HC, AD and VaD groups.

* p < 0.05 between HC and AD or VaD;

† *p < 0.05 between AD and VaD*.

## Results

**Figure 1:**
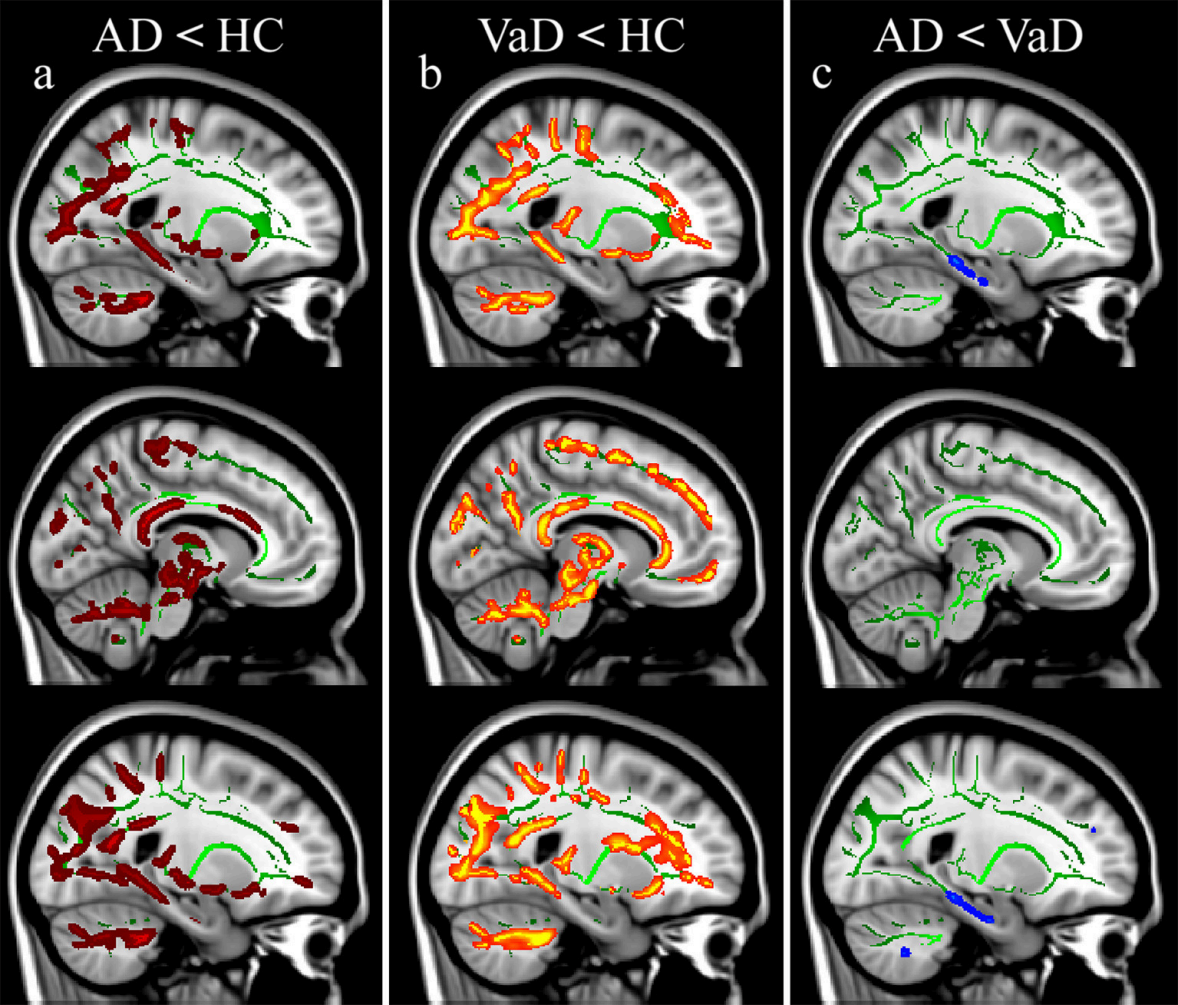
Fractional anisotropy (FA) alterations in patients. Significance was set at *p* < 0.01 TFCE corrected for multiple comparisons. All results are overlaid onto the MNI 152 template and are shown as sagittal slices. Top row: left hemisphere (x = −22 mm). Middle row: medial view (x = −9 mm). Bottom row: right hemisphere (x = 23 mm). FA reductions are reported in: **(a)** AD vs. HC, **(b)** VaD vs. HC, **(c)** AD vs. VaD.

**Figure 2:**
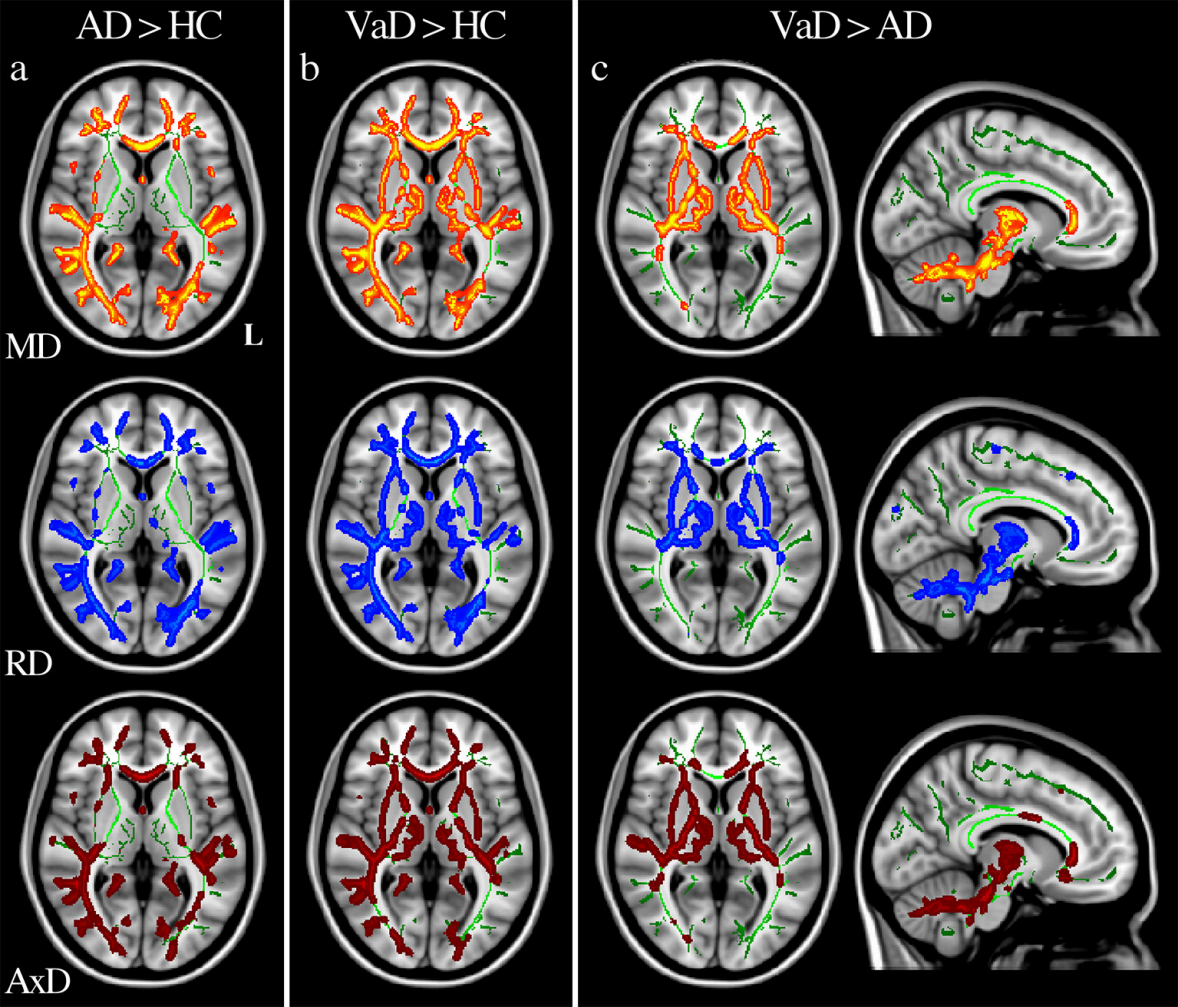
Diffusivity alterations in patients. Significance was set at *p* < 0.01 TFCE corrected for multiple comparisons. All results are overlaid onto the MNI 152 template. Axial slices correspond to z = 4 mm while sagittal slices correspond to x = −9 mm. L = left hemisphere. Increases of MD (top row), RD (middle row), and AxD (bottom row) are reported in: **(a)** AD vs. HC, **(b)** VaD vs. HC, **(c)** VaD vs. AD.

Brain areas with abnormal DTI-derived parameters in patients with respect to HC are summarized in Tables 3–5 and are shown in Figures 1, 2.

**Table 3:** Diffusion changes between HC and AD.

	**AD < HC**	**AD > HC**
FA	CRBL - PhT - CC (body and splenium) **-** ThR - occipital, parietal and temporal lobes	Left CST
MD	None	WCA, except for: ThR, anterior CB and CST
RD	None	WBA, except for: ThR, anterior CB and CST
AxD	None	WCA, except for: ThR, anterior CB and CST

*Regions of significant alterations identified as FA reductions and as MD, RD, and AxD increases. WBA, widespread brain alteration; WCA, widespread cerebrum alteration; CRBL, cerebellum; ThR, thalamic radiation; PhT, parahippocampal tract; CC, corpus callosum; CST, corticospinal tract; CB, cingulum bundle*.

**Table 4:** Diffusion changes between HC and VaD.

	**VD < HC**	**VD > HC**
FA	WBA, except for: anterior PhT, CC (splenium), CB and CST	None
MD	None	WBA, except for anterior right PhT
RD	None	WBA, except for anterior right PhT
AxD	None	WBA, except for anterior right PhT

*Areas of significant alterations identified as FA reductions and as MD, RD, and AxD increases. WBA, widespread brain alteration; PhT, parahippocampal tract; CC, corpus callosum; CST, corticospinal tract; CB, cingulum bundle*.

**Table 5:** Diffusion changes between AD and VaD.

	**AD < VD**	**AD > VD**
FA	Anterior PhT	CRBL - left ThR - SLF
MD	CRBL - ThR - SLF - CC (genu) - CST	Anterior Right PhT
RD	CRBL - ThR - SLF - CC (genu) - CST	Anterior PhT
AxD	CRBL - ThR - SLF - CC (genu) - CST	None

*Areas of significant alterations identified as FA reductions and as MD, RD, and AxD increases. CRBL, cerebellum; ThR, thalamic radiation; PhT, parahippocampal tract; CC, corpus callosum; CST, corticospinal tract; SLF, superior longitudinal fasciculus*.

### Patient characteristics

Demographic, clinical, neuropsychological and radiological characteristics are summarized in Table 2. Age and gender were significantly different in VaD patients with respect to HC and AD. MMSE and years of education were significantly reduced in patients with respect to HC, but they did not differ between AD and VaD. Clinical and radiological scores, namely Hachinski and Fazekas, were significantly higher in VaD than in AD.

### Changes in AD compared to HC

AD patients compared with HC showed a reduction of FA predominantly in the posterior part of occipital, parietal and temporal lobes, while a small portion of the left corticospinal tract showed increased FA values. Decreased FA values were revealed bilaterally in the cerebellum (lobules VI, VIII, IX and Crus), parahippocampal tracts, thalamic radiations, and body and splenium of corpus callosum (Figure 1a). Moreover, compared with HC, AD patients showed an increase of MD, RD, and AxD in widespread brain regions, except for bilateral thalamic radiations (Figure 2a), anterior cingulum bundles and corticospinal tracts. The cerebellum showed increased RD in the Crus and lobules VIII and IX. No areas of decreased MD, RD, and AxD values were detected in AD patients with respect to HC (see Table 3).

### Changes in VaD compared to HC

VaD patients compared with HC showed significantly reduced FA values widespread in most of the WM except for bilateral anterior parahippocampal tracts, cingulum bundles, corticospinal tracts and splenium of corpus callosum (Figure 1b). Increased MD, RD, and AxD was found in most of the WM except for the anterior part of the right parahippocampal tract. No areas of increased FA or decreased MD, RD, and AxD were found in VaD patients with respect to HC subjects (see Table 4).

### Direct comparison of AD and VaD

AD patients compared with VaD patients showed FA reduction and RD increase in bilateral anterior parahippocampal tracts (Figure 1c), while higher MD values were found only in the right anterior parahippocampal tracts. No areas of increased AxD were found in AD compared with VaD patients (see Table 5).

VaD patients compared with AD patients showed FA reductions and diffusivity increases in the posterolateral cerebellar area (Figure 2c), left thalamic radiation and bilateral superior longitudinal fasciculi. MD, RD, and AxD increases were also found in right thalamic radiation, genu of corpus callosum (Figure 2c) and bilateral corticospinal tracts (see Table 5).

### Discriminative power of DTI parameters between the VaD and AD groups

**Figure 3:**
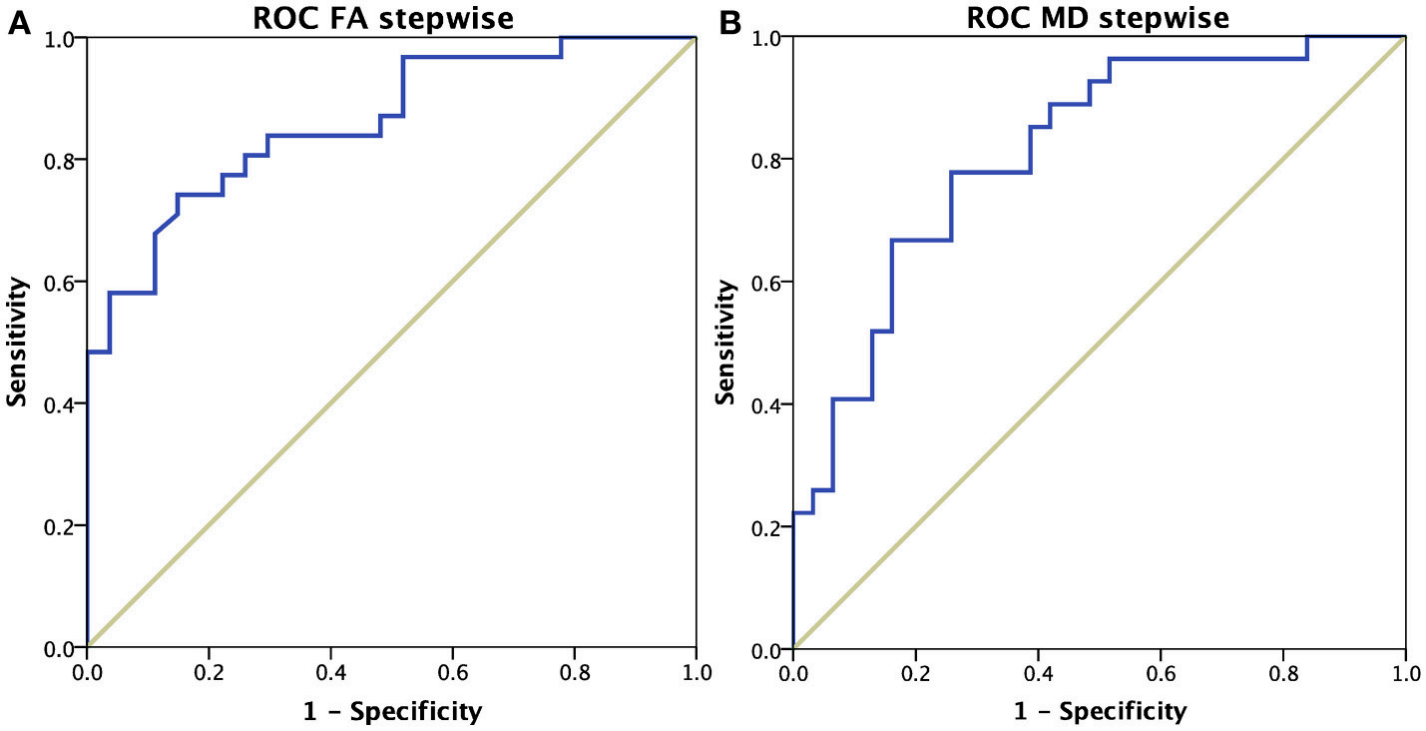
ROC curves of dementia subgroups differentiation. **(A)** Results using mean FA values of five brain regions: left parahippocampal tract, right cingulum, genu of the corpus callosum, and bilateral anterior thalamic radiations. **(B)** Results using mean MD values of two brain regions: left parahippocampal tract and right anterior thalamic radiations.

The combination of mean FA values of five regions, including the left parahippocampal tract, the right cingulum, the genu of the corpus callosum, and bilateral anterior thalamic radiations best discriminated between AD and VaD (Wilks' lambda = 0.542, *p* < 0.001). The average squared canonical correlation was 0.677, showing that these five variables, accounted for 67.7% of the overall variance in the data set. The discriminative function equation (composite score) was as follows: – 0.584 ^*^ left parahippocampal tract – 0.619 ^*^ right cingulum + 1.253 ^*^ genu of the corpus callosum – 1.879 ^*^ right anterior thalamic radiation + 2.033 ^*^ left anterior thalamic radiation. The ROC curve (Figure 3A) showed a 75.9% overall correct classification in discriminating AD and VaD, with a sensitivity of 74.2%, specificity of 77.8%, and area under the curve of 0.861.

The discriminative power of mean MD values achieved its maximum when combining two brain areas: the left parahippocampal tract and the right thalamic radiation (Wilks' lambda = 0.724, *p* < 0.001). The average squared canonical correlation was 0.525, showing that these two variables, accounted for 52.5% of the overall variance in the data set. The discriminative function equation (composite score) was as follows: – 0.662 ^*^ left parahippocampal tract + 1.018 ^*^ right anterior thalamic radiation. The ROC curve (Figure 3B) showed a 74.1% overall correct classification in discriminating AD and VaD, with a sensitivity of 66.7%, specificity of 80.6%, and area under the curve of 0.810.

### Correlations

Pearson correlation verified that worse DTI derived parameters (i.e., smaller FA and higher MD values) were related with higher vascular impairment (i.e., higher Fazekas and ARWMC values) (Tables 6, 7) and that total Fazekas score correlated with deficit of the memory domain (*p* = 0.002) in VaD patients.

**Table 6:** Fazekas correlations with DTI derived parameters.

		**Patients (*n* = 58)**	**AD (*n* = 31)**	**VaD (*n* = 27)**
**FA**	CC genu	<0.001	0.005	–
	CC anterior body	<0.001	0.006	0.007
	CC posterior body	0.002	0.002	–
	CC splenium	0.01	0.010	–
	Thal radiation right	<0.001	0.009	–
	Thal radiation left	<0.001	–	–
	Parahipp tract left	–	0.005	–
**MD**	CC genu	<0.001	–	–
	CC anterior body	<0.001	–	0.007
	CC posterior body	<0.001	0.002	–
	CC splenium	0.001	0.01	0.005
	Thal radiation right	<0.001	0.001	0.007
	Thal radiation left	<0.001	<0.001	–
	Cingulum right	0.001	0.005	–
	Cingulum left	<0.001	0.010	–

*Pearson correlations between Fazekas score and DTI-based parameters for all patients, Alzheimer's disease (AD), and vascular dementia (VaD) patients. CC, corpus callosum. Significance was set at p < 0.01*.

**Table 7:** ARWMC basal ganglia correlations with DTI derived parameters.

		**Patients (*n* = 58)**	**AD (*n* = 31)**	**VaD (*n* = 27)**
**FA**	CC genu	<0.001	0.009	–
	CC anterior body	0.001	0.002	–
	CC splenium	0.008	0.01	–
	Thal radiation right	<0.001	–	0.008
	Thal radiation left	0.001	–	–
**MD**	CC genu	0.001	–	–
	CC anterior body	0.001	–	–
	CC posterior body	0.006	–	–
	CC splenium	0.001	0.010	–
	Thal radiation right	<0.001	0.001	0.001
	Thal radiation left	<0.001	0.001	–
	Cingulum right	0.002	–	–
	Cingulum left	<0.001	–	<0.001

*Pearson correlations between ARWMC score and DTI-based parameters for all patients, Alzheimer's disease (AD), and vascular dementia (VaD) patients. CC, corpus callosum. Significance was set at p < 0.01*.

MMSE did not correlate with any of the MRI derived measurements, while attention, language, executive functions, and visuo-spatial skills negatively correlated with MD values of the corpus callosum, cinguli and left parahippocampal tract of all patients (details in Table 8). In particular, attention and language deficits were mainly associated with VaD alterations while executive functions and visuo-spatial skills were mainly associated with AD impairment.

**Table 8:** Neuropsychological correlations with DTI derived parameters.

		**Patients (*n* = 58)**	**AD (*n* = 31)**	**VaD (*n* = 27)**
Attention	MD cingulum left	–	–	0.020
	MD CC genu	0.017	–	–
	MD CC anterior body	0.026	–	–
	MD CC splenium	0.034	–	–
Language	MD Parahipp tract left	0.030	–	–
	MD cingulum left	–	–	0.048
	MD CC splenium	–	–	0.020
Executive function	MD Parahipp tract left	0.042	0.046	–
	MD CC splenium	0.016	–	–
Visuo-spatial skills	MD cingulum right	–	0.045	–
	MD CC splenium	0.007	0.027	–

*Pearson correlations between neuropsychological tests and DTI-based parameters for all patients, Alzheimer's disease (AD), and vascular dementia (VaD) patients. MD, mean diffusivity; CC, corpus callosum. Significance was set at p < 0.05*.

## Discussion

The present study is one of the few studies that directly compares AD with VaD patients in a global framework using a standardized acquisition protocol and a voxelwise user-independent analysis. Both anisotropy (FA) and diffusivity (MD, AxD, and MD) parameters were investigated in terms of changes due to either alterations of anisotropy or magnitude of water diffusion in the whole brain (Shu et al., 2011). In general, it can be argued that diffusion alterations could be mainly interpreted as associated with tract disconnection in AD patients, while diffusivities increases are correlated with leukoariosis and WM lesions highly present in VaD patients, highlighting that AD and VaD show specific patterns of alteration and are indeed different pathologies (Alves et al., 2015; Palesi et al., 2016). Furthermore, it is important to note that age and gender can influence white matter measurements, hence groups matched for age and gender would be preferred. When this condition is not satisfied, as in the present study, it is necessary to use complex statistical models with covariates, such as multivariate regressions, to compare groups. In this context, the present study identifies several regions that can be useful in distinguishing between AD and VaD patients, providing information regarding specific WM alteration processes involved in the two different pathologies.

It was very interesting to detect specific regional changes affecting predominantly AD or VaD, defining a pathology-specific signature, which we summarize here before discussing in detail each pathology in turn and the discriminative analysis. The parahippocampal tracts resulted to be distinctive between AD patients and HC, confirming previous histopathological (Detoledo-Morrell et al., 1997; Salat et al., 2010; Echávarri et al., 2011), and DTI-based findings (Palesi et al., 2012; Acosta-Cabronero and Nestor, 2014). Our regional results confirmed that the anterior part of the parahippocampal tracts was the most affected region in AD also as compared with VaD. This is not surprising knowing the central involvement of the hippocampus in AD (Vitali et al., 2008), nevertheless this alteration directly between AD and VaD has not been previously reported. Furthermore, we detected a high specificity in the involvement of sub-regions of the corpus callosum, where different patterns of decreased FA characterized either pathology: the splenium was mainly affected in AD, while the genu was mainly affected in VaD patients. This differential involvement of the corpus callosum is consistent with known pathogenesis of the two dementia types. Indeed, the hippocampus/precuneus circuit, which includes the parahippocampal tract, posterior cingulum and the splenium of the corpus callosum, is affected quite early in AD (Villain et al., 2010; Palesi et al., 2012), while the genu of corpus callosum connects the two hemispheres of frontal WM that are the main leukoaraiosis areas in VaD. DTI-parameters of thalamic radiations also distinguish between AD and VaD: MD, RD, and AxD increases were indeed found only in VaD (both vs.AD and HC) but not in AD.

The discriminative power of these findings was quantitatively confirmed with a step-wise discriminative analysis that revealed how diffusion values of these regions were able to distinguish subtypes of dementia with an accuracy of 75% (sensitivity of 70%, specificity of 78%). Furthermore, the Pearson correlation analysis identified a direct relationship between vascular load, quantified by Fazekas and ARWMC scores, and degeneration of the thalamic radiation, callosal and parahippocampal tracts. Therefore, diffuse microstructural abnormalities identified by DTI are tightly related to multifocal lesions (lacunae and leukoariosis), not only in VaD but also in AD.

### AD alterations

Parahippocampal tracts and corpus callosum mainly showed microstructural degeneration in AD. Indeed, both FA decreases (Figure 1a) and diffusivity increases (Figure 2a) characterized the parahippocampal tracts when AD and HC were compared, while only FA decreases (Figure 1c) and RD increases were found in bilateral anterior parahippocampal tracts when AD and VaD were compared. MD increased in the right parahippocampal tract, while the left tract showed a less significant (*p* < 0.05) MD increase. In accordance with previous studies (Palesi et al., 2012; Acosta-Cabronero and Nestor, 2014), our DTI-derived parameters detect microstructural abnormality in parahippocampal tracts in early stage of AD, thus indicating they are promising candidates as neuroimaging markers to characterize AD patients and differentiate them from VaD.

Furthermore, the corpus callosum was characterized by an increase in all diffusivity parameters (MD, RD, and AxD) and a decrease in FA mainly in its posterior part (Figure 1a) when comparing AD with HC. Pathophysiological implications of corpus callosum alterations in AD have already been discussed (Takahashi et al., 2002; Stricker et al., 2009; Acosta-Cabronero et al., 2010, 2012; O'Dwyer et al., 2011). Corpus callosum impairment would lead to a disconnection between the two hemispheres and consequently to cognitive impairment due to a disruption of communication between interconnected networks (Genc et al., 2015).

Several other regions were involved in AD. Thalamic radiations showed only decreased FA, without changes in diffusivity parameters in AD with respect to HC. These results have been previously explained as a consequence of an involvement of thalamo-cortical pathways (Medina et al., 2006; Rose et al., 2008; Salat et al., 2010; Zarei et al., 2010). The degeneration of the posterior part of the bilateral cingulum was revealed by increased diffusivity parameters (MD, RD, and AxD) and, in accordance with previous studies (O'Dwyer et al., 2011; Acosta-Cabronero et al., 2012; Bosch et al., 2012), has been interpreted as one of the principal factors leading to the cognitive and memory decline in AD (Gili et al., 2011; Suri et al., 2014). Indeed, the cingulum is a crucial part of the limbic system, and one of the major WM fasciculi connecting associative cortical areas (Bozzali et al., 2012), implying that damage of the cingulum would result in a diffuse “disconnection” syndrome. Cognitive impairment was also supported by FA decrease and RD increase in the cerebellar posterolateral regions, because these regions are directly connected with cerebral associative areas (Stoodley and Schmahmann, 2010; Palesi et al., 2015, 2017). Previous TBSS works have reported cerebellar involvement in AD (Acosta-Cabronero et al., 2010; Liu et al., 2011; Teipel et al., 2014), which has been also shown by using different MRI modalities (Wang et al., 2007; Castellazzi et al., 2014).

It is also worth noting that increased FA was detected in the corticospinal tracts of AD with respect to HC group. A possible explanation has been given by Teipel et al. (2014), who suggested that higher FA in the corticospinal tracts is associated with an increase in the Mode of anisotropy, which reflects shape characteristics of the diffusion tensor (Ennis and Kindlmann, 2006). The simultaneous increase in both FA and Mode could mirror a loss of crossing fibers rather than an actual increment of diffusivity along high-coherence fiber bundles. Consequently, higher FA may be a piece of evidence of a relative sparing of the corticospinal tract in the presence of degeneration of other fibers crossing the pyramidal tract. Analysis of fiber orientation dispersion indices may be helpful in disentangling the sources of such anisotropy changes.

### VaD alterations

Although widespread areas of alterations emerged from the analysis (see Tables 4, 5 for details), our results were also able to identify a few regions specific to VaD patients. Such difficulty in finding specific patterns of WM alterations in VaD has been also encountered in previous studies that reported FA reductions in widespread brain regions in VaD, both in comparison with AD and HC (Sugihara et al., 2004; Mayzel-Oreg et al., 2007; Zarei et al., 2009; Suri et al., 2014). However, to our knowledge only one study directly compared all DTI-derived parameters between VaD, AD and HC (Suri et al., 2014).

The genu of corpus callosum showed decrease FA in VaD with respect to HC (Figure 1b), while diffusivity (MD, RD and AxD) increases were found in the same region with respect to AD (Figure 2c). These findings are in agreement with others (Zarei et al., 2009; Fu et al., 2012; Suri et al., 2014; Wu et al., 2015) and support the hypothesis that impairment in the genu of corpus callosum in VaD leads to a loss of inter-hemispheric prefrontal connections with a consequent executive dysfunction.

Furthermore, increases in MD, RD and AxD and decrease FA were found in thalamic radiations and in posterior cerebellar lobules of VaD patients with respect to HC and AD (Tables 4, 5, Figures 2b,c). No DTI-based studies have reported such diffuse involvement of these white matter tracts, nevertheless thalamic lesions are known to be associated with VaD and to cause cognitive impairment (Cummings, 1994; Szirmai et al., 2002), while cerebellar alterations suggest a more severe cerebellar-mediated cognitive impairment in VaD than in AD patients.

FA reduction and diffusivity increase were also found in superior longitudinal fasciculi, which is the main hemispheric tract connecting fronto-parietal regions, and corticospinal tracts of VaD patients with respect to AD (Table 5). DTI abnormalities in the superior longitudinal fasciculi have been previously reported in VaD (Fu et al., 2012), while there are no reports of differences in the corticospinal tracts that can be related to the subtle subclinical pyramidal deficits in VaD (Foster et al., 2014).

### Differentiating between dementias: direct comparison of AD and VaD

Our results demonstrated that only FA identified specific alteration patterns between subtypes of dementia and healthy control subjects. On the contrary, other DTI-parameters revealed specific clusters of alteration, as opposed to widespread impairment, only when AD and VaD were directly compared.

The WM regions showing alterations specific to each type of dementia were the parahippocampal tracts and the corpus callosum. In these tracts it was possible to identify an anterior—posterior gradient of changes. Alterations of the anterior part of parahippocampal tracts were specific to AD while posterior alterations were found in both dementia groups. Indeed, VaD showed diffusivity increases in widespread brain regions but not in the anterior parahippocampal tracts that were spared (Figure 1b). Similar results were also discussed by Kim et al. (2015) who reported FA alterations mainly in the fronto-parietal WM regions of AD patients, while only a few specific WM structures were preserved in VaD. In our AD patients, FA values of the left parahippocampal tract directly correlated with the Fazekas score suggesting a vascular contribution to the AD pathogenesis. Furthermore, FA reductions characterized the splenium of corpus callosum in AD patients while all DTI-parameters were altered in the genu of corpus callosum in VaD patients (Figures 1, 2) suggesting that the corpus callosum is more impaired in VaD than in AD. These findings confirmed those by Zarei et al. (2009), which identified the corpus callosum as the most important structure to differentiate different types of dementia. Furthermore, Zarei et al. (2009) reported the importance of integrating FA values with vascular indices, e.g., the Fazekas score, to characterize subtypes of dementia. In this contest, our Pearson correlation analysis confirmed the vascular involvement of the corpus callosum in all patients and revealed that the anterior part of the corpus callosum was mainly involved in VaD patients.

Furthermore, our results suggest that also the thalamic radiations are potentially useful for distinguishing between AD and VaD. Indeed, only VaD patients showed changes in MD, RD and AxD of the thalamic radiations with respect to HC (Figures 2b,c). Interestingly, such structure was shown to be affected in VaD also when compared with AD; therefore, it could be argued that an increase in diffusivity of the thalamic radiations is linked to tracts disconnection and plays an important role in distinguishing VaD from neurodegenerative dementia. This is an innovative result, in fact none of the previous TBSS studies has reported evidence of specific thalamic alterations useful to differentiate these types of dementia. The importance of microstructural changes in the thalamic radiations is further supported by its correlation with general and deep vascular load both in AD and VaD patients, which could highlight the key role of the vascular contribution for developing dementia in both groups.

When the clinical picture is uncertain, searching for an anterior-to-posterior lesional pattern of alterations in the parahippocampal tracts and corpus callosum, alongside with changes in diffusivity magnitude in the thalamic radiations would be helpful to clarify the diagnosis. We are aware that clinical application of the present study is not immediate and further studies are warranted to confirm these results but we are confident that our findings would be a good starting point for developing a machine learning approach to help the diagnostic workup.

### Methodological considerations

WM lesions are present in VaD, but also in AD patients. Such lesions can indeed influence the evaluation of DTI parameters and consequently the statistical results of comparisons between groups. No previous TBSS study has taken into account this aspect. VaD patients mainly show lesions randomly located throughout the whole WM, and consequently it is difficult to obtain a mean lesion probability map. However, most of our results are in accordance with previous reports. In addition, alteration patterns that were identified as the most specific to each type of dementia were located in WM structures known to be involved in AD and VaD pathologies. Future studies should develop ways to incorporate lesional information in TBSS analysis for dementia and other neurological and neurodegenerative pathologies.

A limitation of the present study is the relatively small cohort of subjects. Indeed, the use of a larger cohort would provide a better clinical characterization of the patient groups and may identify significant correlations between the neuropsychological profile of patients and their MRI-derived parameters. This would be useful for distinguishing subtypes of dementia from both a structural and a clinical point of view and for supporting clinical diagnosis.

The implications of the present findings in cases of mixed dementia are unclear. In this study, patients with mixed dementia were excluded in the initial phase of the enrollment, based on the clinical and neuropsychological assessment along with the administration of the Hachinski scale. This tool let us identify two relatively pure etiologic groups (AD and VaD). Therefore, the relative weight of the MRI changes observed in this study in defining as prevalent the nature (neurodegenerative vs. vascular) of the dementing process in these patients cannot be ascertained according to our data. Specifically designed studies in patients with mixed dementia should address this important issue.

## Conclusions

Alteration patterns associated to disconnection of specific tracts and useful in distinguishing between AD, VaD, and HC were found using an advanced DTI sequence acquired on a 3T scanner. The method presented here cannot be proposed at present as a substitute for classical diagnostic tools, but rather could provide a mean to map the degree of circuit disconnection of different types of dementia and to discriminate between AD and VaD. DTI metrics may thus be incorporated in the multimodal staging of dementia, since these results can be combined with other MRI data (functional MRI, spectroscopy) and other tools [cerebrospinal fluid examination, positron emission tomography (PET)] to obtain a further confirmation of diagnosis and progression. The overarching aim of future studies should be to gather microstructural information in addition to a battery of clinical, radiological and neuropsychological tests, as well as other quantitative imaging metrics, to develop an automatic classification approach improving sensitivity of the diagnostic process. The use of multiple DTI indices in addition to other standard multimodal methods would help to differentiate the contribution of vascular load and primary neurodegeneration to the evolution of cognitive disturbances in AD and VaD.

## Author contributions

FP, AD, CGW-K, and ED conceptualized the study. AD and FP designed and performed the analyses with support from GC and LC. GG, PV, and NA acquired all MRI data. PV and NA performed semi-quantitative rating of vascular abnormalities. ES and SB acquired all neuropsychological data helping for data interpretation. AC, MR, FD, and GMi enrolled all patients and performed all clinical evaluations. CGW-K, ED, and GMa provided support and guidance with data interpretation with clinical contribution of all physicians. FP, AD, PV, CGW-K, and ED wrote the manuscript, with comments from all other authors.

### Conflict of interest statement

The authors declare that the research was conducted in the absence of any commercial or financial relationships that could be construed as a potential conflict of interest.

## Abbreviations

AD: Alzheimer's disease
AxD: axial diffusivity
DTI: diffusion tensor imaging
FA: fractional anisotropy
MD: mean diffusivity
MMSE: Mini-Mental State Examination
RD: radial diffusivity
TBSS: tract-based spatial statistics
VaD: vascular dementia.
