# Supplementary figures and images for: Specific Patterns of White Matter Alterations Help Distinguishing Alzheimer's and Vascular Dementia
Source: Front Neurosci. 2018 Apr 25;12:274. doi: 10.3389/fnins.2018.00274 (PMC5996902; doi:10.3389/fnins.2018.00274)

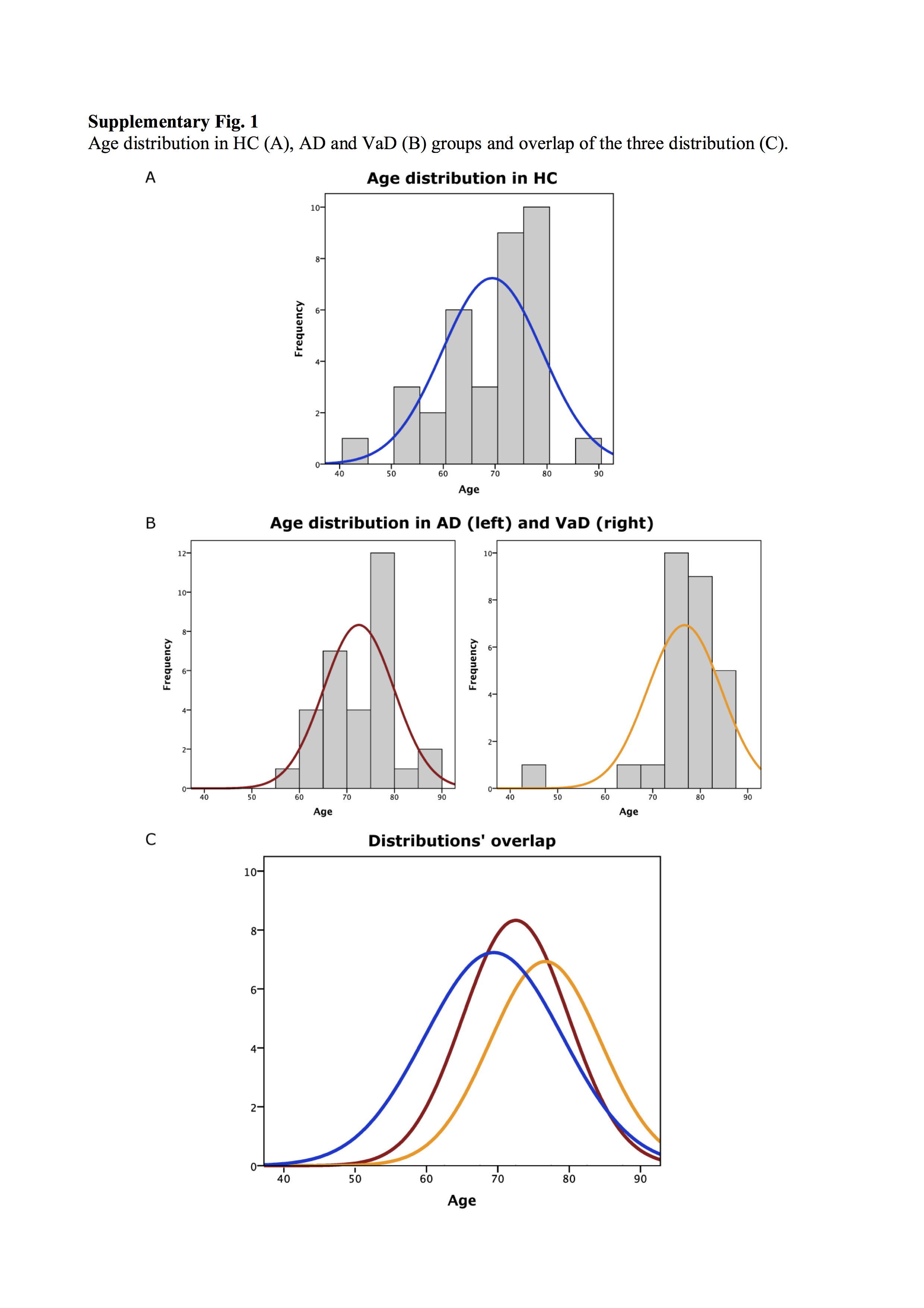

Supplement: Supplementary file 1 [file Image_1.tiff]
